# Bile Acid Composition and Transcriptome Analysis of the Liver and Small Intestine in Different Species

**DOI:** 10.3390/metabo14080451

**Published:** 2024-08-15

**Authors:** Dongming Qi, Tingting Zheng, Maosen Yang, Zhiying Huang, Tao Wang, Qiang Wang, Binlong Chen

**Affiliations:** 1 College of Animal Science, Xichang University, Xichang 615000, China; qidongming1982@163.com; 2 School of Life Sciences, China West Normal University, Nanchong 637009, China; ztt12172024@163.com; 3 State Key Laboratory for Conservation and Utilization of Subtropical Agrobioresources, College of Animal Science and Technology, Guangxi University, Nanning 530004, China; youngmaosen@126.com; 4 College of Animal Science, Shanxi Agricultural University, Taiyuan 030801, China; huangzhiying2000@163.com; 5 School of Pharmacy, Chengdu University, Chengdu 610106, China; wangtao3@cdu.edu.cn; 6 Ningnan County Bureau of Agriculture and Rural Affairs, Xichang 615400, China; haisenselove@163.com

**Keywords:** different species, BA content, mRNA, correlation analysis

## Abstract

Bile, a crucial fluid produced continuously by the liver, plays an essential role in digestion within the small intestine. Beyond its primary function in lipid digestion, bile also acts as a pathway for the elimination of various endogenous and exogenous substances. There have been limited studies focusing on interspecies differences. This study offers a comprehensive analysis of bile acid (BA) composition and its correlation with gene expression patterns across six different species, including mammals and poultry, through combining Liquid Chromatography-Mass Spectrometry (LC-MS) and transcriptome sequencing. The BA profiles revealed distinct metabolite clusters: D-glucuronic acid (GLCA) and glycochenodeoxycholic acid (GCDCA) were predominant in mammals, while taurolithocholic acid (TLCA) and T-alpha-MCA were prevalent in poultry, highlighting species-specific BA compositions. Differentially abundant metabolites, particularly GDCA, glycohyodeoxycholic acid (GHDCA) and taurodeoxycholic acid (TDCA) showed significant variations across species, with pigs showing the highest BA content. Transcriptome analysis of the liver and small intestine tissues of 56 cDNA libraries across the six species revealed distinct mRNA expression patterns. These patterns clustered samples into broad categories based on tissue type and phylogenetic relationships. Furthermore, the correlation between gene expression and BA content was examined, identifying the top 20 genes with significant associations. These genes potentially serve as biomarkers for BA regulation.

## 1. Introduction

Bile acids (BAs), produced in the liver, undergo extensive metabolic changes before being secreted into enterohepatic and systemic circulation [1]. The composition of bile from the liver is predominantly water, which comprises about 95% of its volume. The remaining components include bile acids, bilirubin, lipids, a small fraction of proteins, and other metabolic byproducts [2]. Recently, the potential value of bile analysis has been increasingly recognized, promoting a growing number of studies in this area. It is hypothesized that alterations in the bile acid levels may indicate pathological changes within the body [3]. This hypothesis has driven further investigation into the role of bile and its components as potential biomarkers for various health conditions.

The liver plays a critical role in the digestive and immune systems [4]. In the liver, cholesterol is transformed into two primary bile acids, cholic acid (CA) and chenodeoxycholic acid (CDCA), which are then conjugated to glycine or taurine through bile acid synthetic pathways [5]. Over the last two decades, bile acids have been recognized as pivotal signaling molecules in the pathogenesis of nonalcoholic fatty liver disease, enabling fine-tuned gut–liver communication [6]. This communication occurs from the liver, which produce BAs, to the intestine, the site of nutrition sensing, and extends throughout the body, where bile acids exert their pleiotropic physiological functions. Bile acids are a collection of molecules with both hydrophilic and hydrophobic properties. They are synthesized by hepatocytes from cholesterol and are released via the bile ducts into the digestive tract. These molecules play a crucial role in the emulsification of fats, as well as in the digestion and absorption of lipids and fat-soluble vitamins following the consumption of food [7]. Consequently, gene expressions in the liver and intestine are closely related to the production of BAs.

Bile acids are absent from invertebrates [8], but present in all vertebrate species [9]. As a type of animal medicine, bile from cattle and other animals has anti-inflammatory, antipyretic and cholagogic effects [10]. However, limited research has been conducted to explore the differences in animal bile among species, as well as their gene expression patterns in the liver and small intestine. The combined use of transcriptomics and Liquid Chromatography-Mass Spectrometry (LC-MS) in different species offers a multi-dimensional understanding of liver and intestinal function in bile acid’s formation and metabolism. Transcriptomics provides insights into gene expression patterns. In contrast, LC-MS offers a precise analysis of bile acids, crucial for digestion and metabolic processes. By integrating these approaches across species, we can identify conserved and species-specific mechanisms, enhancing our understanding of physiology differences among species. It is easy to obtain bile acids from domestic animals (like pigs and geese). The comparative perspective is invaluable for identifying evolutionary adaptations, unraveling disease mechanisms according to their different phenotypes, and discovering potential therapeutic targets, ultimately benefiting translational medicine and pharmacology with BAs. To date, no study has investigated the association between transcriptome profiles and bile acid compositions in commonly domesticated animals, especially within the two key tissues (liver and small intestine) that play a significant role in bile acid metabolism. Using the six most frequent domesticated livestock species (sheep, cattle, pigs, chickens, ducks, and geese) raised in China, the goal of the current study was to utilize bile acid metabolomics and liver and small intestine transcriptomics to gain insight into the differences among species.

## 2. Materials and Methods

### 2.1. Ethics Statement

All methods were performed in accordance with the relevant guidelines and regulations provided by the Regulations of the Administration of Affairs Concerning Experimental Animals (China, 1998) for animal experiments. All experiments were reviewed and approved by the Committee for the Care and Use of Laboratory Animals of Xichang University on 25 December 2023 (Approval ID: 20231225). Maximal effort was applied in order to minimize the suffering of the animals.

### 2.2. Experimental Animals and Samples

This study examined six types of animals with different molecular analyses, including the Yanying chicken, Jianchang Duck, Gang goose, Xichang black pig, Tibetan sheep, and Simmental cattle. All experimental animals were female, with chickens, ducks, and geese selected at the age of 200 days and pigs, cattle, and sheep selected at the age of 2.5 years. The chickens, ducks, and geese were kept on the same farm (Xichang Huining Agriculture and Animal Husbandry Technology Co., Ltd., Xichang, China), with free ranging, and were allowed to eat corn and drink water freely. The cattle and sheep were kept on the same farm (Xichang Wanke Yangguang Ecological Cultivation and Planting Professional Cooperative, Xichang, China), with free ranging and free access to grazing and water. The pigs were kept on Xichang Yi Wanjia Family Farm and raised with feeding standard in China (GB/T 5915-2020) [11]. Tissues were collected in the morning, following euthanasia, and included the liver, small intestine, and bile. These samples were collected from 4 chickens, sheep, and pigs, 5 ducks and geese, and 6 cattle. Cervical dislocation or disarticulation of the skull and cervical vertebrae was used for the euthanasia of the three poultry species [12]. For the three mammal species, all the animals were slaughtered via electrical stunning followed by exsanguination [13]. Immediately after collection, tissues were thoroughly washed with PBS and perfused, followed by biospecimen sample storage at −80 °C. Bile was used for bile acid (BA) analysis, while the liver and small intestine were used for transcriptome analysis.

### 2.3. Metabolome Processing and Analyses

Standard solution preparation followed a standard procedure, including weighing of the bile standard and dissolving it with methanol to make a final concentration of 1000 μg/mL standard stock solution, which was then diluted using 30% methanol. All stock solutions and working standard solutions were stored at −20 °C.

Sample preparation: samples were extracted using liquid/liquid extraction. Samples were extracted in 600 μL of methanol (−20 °C), vortexed for 60 s, and centrifuged at 12,000 rpm at 4 °C for 10 min. Then, 30% methanol was used to dilute an appropriate amount of supernatant to 50 times the original volume. The supernatant was filtered through a 0.22 μm membrane, and the filtrate was added to the LC-MS bottle [14].

Metabolome analyses: metabolite levels were measured from bile samples, using a combination of direct injection mass spectrometry with a reverse-phase LC–MS/MS.

Liquid chromatography conditions: an ACQUITY UPLC^®^ BEH C18 column (2.1 × 100 mm, 1.7 μm, Milford, CT, USA) was used, with an injection volume of 5 μL; the column temperature was maintained at 40 °C, and the mobile phase was A—0.01% formic acid water, B—acetonitrile. The gradient elution conditions were 0~4 min, 25% B; 4~9 min, 25~30% B; 9~14 min, 30~36% B; 14~18 min, 36~38% B; 18~24 min, 38~50% B; 24~32 min, 50~75% B; 32~33 min, 75~90% B; 33~35.5 min, 90~25% B; and 35.5~37 min, 25% B. The flow rate was 0.3 mL/min [15,16].

Mass spectrum conditions: electrospray ionization (ESI) source and negative ionization mode. The ion source temperature was 500 °C, the ion source voltage was −4500 V, the collision gas was 6 psi, the curtain gas was 30 psi, and the atomizing gas and auxiliary gas were both 50 psi. Scans were performed using multiple reaction monitoring (MRM) [15,16].

Statistical analysis: MS Convert software (Proteowizard, v3.0.8789) was applied to convert the “.wiff” format raw data to “.mzML” format, which was used for downstream analysis. The software Analyst (v1.7) was used to collect the raw data and qualitatively and quantitatively analyze the results. To assess the technical precision of each experiment, the relative standard deviation of peak areas was calculated for every compound detected in the QC sample (RSD = 100 × standard deviation/average of peak areas), with ideal RSD < 15%. Calibration curves were obtained as plots of the peak area of the target compounds versus the target compound concentration. The peak areas of the target compounds were put into the formula to calculate the concentration in each sample. For calculations, all metabolite concentrations less than 0 were reported as not detected (ND). Only metabolites present in >50% of the samples were kept for further analysis [17]. The T test or a Mann–Whitney–Wilcoxon test was used to calculate *p* values. Differences with *p* value < 0.05 and VIP > 1 were considered statistically significant.

Pathway analysis: target metabolites were subjected to pathway analysis. The identified metabolites in metabolomics were then mapped to Kyoto Encyclopedia of Genes and Genomes (KEGG) pathways for a biological interpretation of higher-level systemic functions. The metabolites and corresponding pathways were visualized using the KEGG Mapper tool [18].

### 2.4. RNA Extraction and Sequencing

Total RNA from liver and small intestine samples was extracted using the TRIzol reagent, according to the manufacturer’s protocol. DNA was removed from each sample using RNase-free DNase. Total RNA from 56 samples was used as the input material for RNA-seq. Briefly, mRNA was purified from total RNA using poly-T oligo-tagged magnetic beads (New England Biolabs, Ipswich, MA, USA). Fragmentation was carried out using divalent cations under an elevated temperature in First-Strand Synthesis Reaction Buffer (New England Biolabs, Ipswich, MA, USA). First-strand cDNA was synthesized using random hexamer primers and M-MuLV Reverse Transcriptase (RNase H-). Second-strand cDNA synthesis was subsequently performed using DNA Polymerase I and RNase H (New England Biolabs, Ipswich, MA, USA). A total of 56 poly-A RNA-Seq libraries were constructed, and a paired-end sequencing length of 150 bp (PE150) was performed on the DNBSEQ-T7.

### 2.5. Transcriptome Analysis

The original fluorescence image files obtained from the Illumina platform were transformed to short reads (raw data) by base calling, and these short reads were recorded in FASTQ format [19], which contains sequence information and corresponding sequencing quality information. Sequencing artifacts, including reads containing adapter contamination, low-quality nucleotides, and unrecognizable nucleotides (N), undoubtedly set the barrier for the subsequent reliable bioinformatics analysis. Hence, quality control is an essential step and applied to guarantee the meaningful downstream analysis. We next used Fastp (version 0.23.1) [20] to perform basic filtering to obtain quality raw reads. The steps of data processing were as follows: (1) Discard paired reads if either one of the reads contains adapter contamination. (2) Discard a paired read if more than 10% of bases are uncertain in either read. (3) Discard paired reads if the proportion of low quality (Phred quality < 5) bases is over 50% in either read.

Pearson’s correlations were calculated across 4~6 samples from each tissue and species; among pairwise comparisons of 6 species within each of the two tissues; and among pairwise comparisons of two tissues within each of the 6 species. PCA and t-SNE (t-distributed stochastic neighbor embedding) analysis was carried out using R (version 4.3.1). We generated the neighbor-joining expression-based tree of the samples according to distance matrices composed of pairwise Spearman’s correlations implemented in the R package [21].

We generated gene expression files by applying Cuffquant (part of Cufflinks) to read mapping results, and further applied expression files to Cuffdiff (part of Cufflinks) to detect DEGs between species pairs from 3 mammals and 3 birds within each group of specific tissues and species. Genes with FDR-adjusted *p*-values ≤ 0.05 were taken as DEGs. Genes were converted to human orthologues and assessed by the DAVID [22] webserver for functional enrichment in gene ontology (GO) terms consisting of molecular function (MF) and biological process (BP), as well as the KEGG pathways (Benjamini-adjusted *p* ≤ 0.05).

## 3. Results

### 3.1. Analysis of the Bile Acid Composition of Different Species

Bile acids (BAs) serve dual roles in the body: as digestive fluids that promote the digestion and absorption of lipids, and as excretory fluids that help to eliminate certain metabolites (such as bilirubin and cholesterol) and non-nutrients through liver biotransformation into the intestinal cavity, where they are excreted in the stool [23]. To explore the composition of BAs across different species, we utilized LC-MS methods to obtain the absolute quantification of 39 types of bile acids in 31 samples from six species.

**Figure 1:**
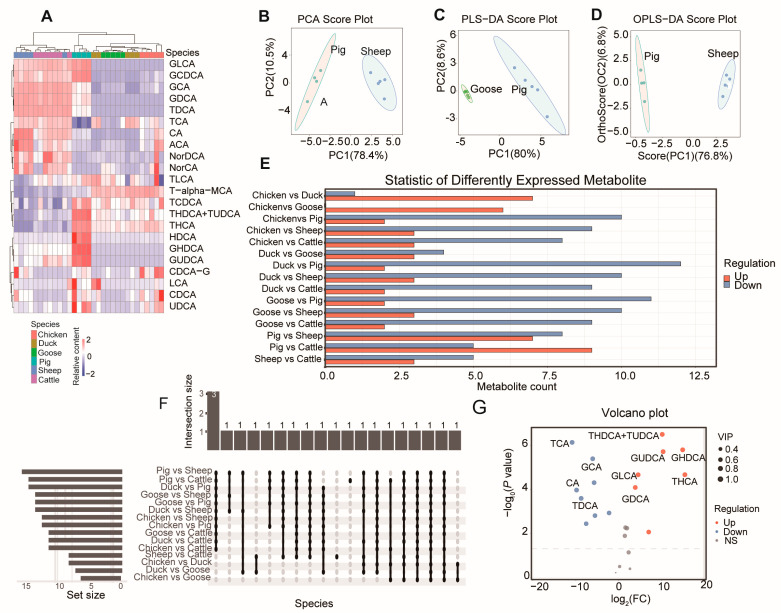
BA composition in different species. (**A**) Agglomerate Hierarchical Clustering and BA content heatmap of the samples among six species. Principal component analysis (PCA) (pigs vs. sheep) (**B**), partial least squares discriminant analysis (PLS-DA) (pigs vs. goose) (**C**), and Orthogonal Projections to Latent Structures Discriminant Analysis (OPLS-DA) (pigs vs. sheep) (**D**) of three representative comparisons. PC1: The first principal component, PC2: the second principal component. Number of differentiated BAs (**E**) and number of overlapped BAs (**F**) between each two species. (**G**) Volcano plot showing the differentiated BAs between pigs and sheep.

As a result, after quality control, the relative standard deviation (RSD) of metabolites in all samples (Figure S1) was less than 15%, indicating high data quality. Using the established metabolite correction curve (Table S1), quantitative calculations were performed for all samples (Table S2). An aggregate clustering heat map showed that these metabolites are clustered according to species. Specifically, GLCA and GCDCA were enriched in mammals (pig, sheep, and goat). Taurochenodeoxycholic acid (TCDCA), Taurohyodeoxycholic acid and Tauroursodeoxycholic acid (THDCA+TUDCA), Tetrahydrocannabinolic (THCA), Hyodeoxycholic acid (HDCA), Glycohyodeoxycholic acid (GHDCA), Glycoursodeoxycholic acid (GUDCA), Chenodeoxycholic acid-3-β-D-glucuronide (CDCA-G), Taurocholic acid (LCA), Chenodeoxycholic acid (CDCA), and UDCA were mainly enriched in pigs. TLCA and T-alpha-MCA had relatively higher content in poultry (including chickens, ducks, and geese). Glycocholic acid (GCA), Chenodeoxycholic acid (GDCA), Taurodeoxycholic acid (TDCA), Taurocholic acid (TCA), Cholic acid (CA), N-(p-amylcinnamoyl) anthranilic acid (ACA), NorDCA, and NorCA were mainly enriched in ruminants (including cattle and sheep) (Figure 1A).

Principal component analysis (PCA) between each pair of species showed that all comparisons had Explainability of the model (R2X) values greater than 0.5 (Table S3). The highest R2X value was found in the comparison between pigs and sheep (Figure 1B). For partial least squares discriminant analysis (PLS-DA) (Figure 1C) and Orthogonal Projections to Latent Structures Discriminant Analysis (OPLS-DA) (Figure 1D), the comparison between geese and sheep had the highest values (both 0.886) (Table S3).

### 3.2. Differentiated BA Metabolites and KEGG Pathway

We next analyzed the differentiated metabolites between each pair of species (Figure 1E), finding the greatest number of differentially expressed metabolites (DEMs) in the comparison between pigs and sheep. Three bile acids (GDCA, GHDCA, and TDCA) showed differences in 11 comparisons (Figure 1F, Table S4). Notably, pigs had the highest bile acid content compared to the same volume in the other five species (Table S5). Compared with sheep, pigs had seven upregulated and eight downregulated DEMs (Figure 1G).

**Figure 2:**
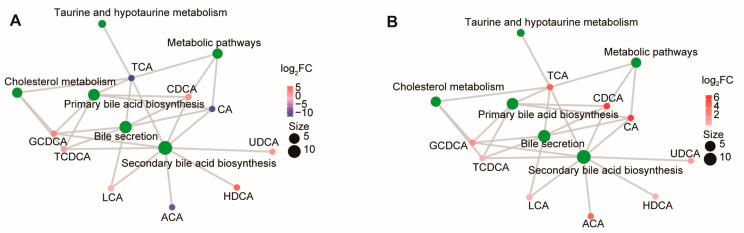
Metabolite pathway analysis. KEGG metabolite molecular network diagram of comparisons performed between sheep and pigs (**A**) and geese and chickens (**B**). Green dots represent metabolic pathways, and other dots represent metabolite molecules. The size of the metabolic pathway point indicates the number of metabolite molecules connected to it, with greater numbers represented by larger points. The metabolite molecular points indicate the size of the log_2_(FC) value through the gradient change.

We then analyzed the KEGG (Kyoto Encyclopedia of Genes and Genomes) pathways enriched with bile acid metabolites. In comparing pigs and sheep, the differentiated BAs were related to “secondary bile acid biosynthesis”, “bile secretion”, “primary bile acid biosynthesis”, “taurine and hypotaurine metabolism”, and “cholesterol metabolism” (Figure 2A). In a comparison between chickens and geese, all differentiated BAs were upregulated in chickens (Figure 2B).

### 3.3. Analysis of the Transcriptome of the Liver and Small Intestine among Different Species

Considering the crucial role of the liver and small intestine in BA synthesis and absorption, we explored the transcriptome pattern of these six species. A total of 56 cDNA libraries (28 liver and 28 small intestine samples) were obtained from four chickens, five ducks, five geese, four pigs, four sheep, and six cattle (Table S6). Sequencing yielded a total of 619.60 Gb of raw data, and the filtered clean data amounted to 592.67 Gb (with an average of 10.57 Gb for each sample), with an average number of 70, 556, 314 clean reads for each sample.

Our RNA-Seq experiments included three mammalian animals (sheep, cattle and pig) and three avian species (chicken, duck and goose), allowing us to track the expression patterns of mRNAs as species-specific processes unfolded. Based on the expression patterns of mRNAs (5801 transcribed single-copy orthologous genes) from the 56 samples, we calculated the Pearson correlation between each pair of samples.

**Figure 3:**
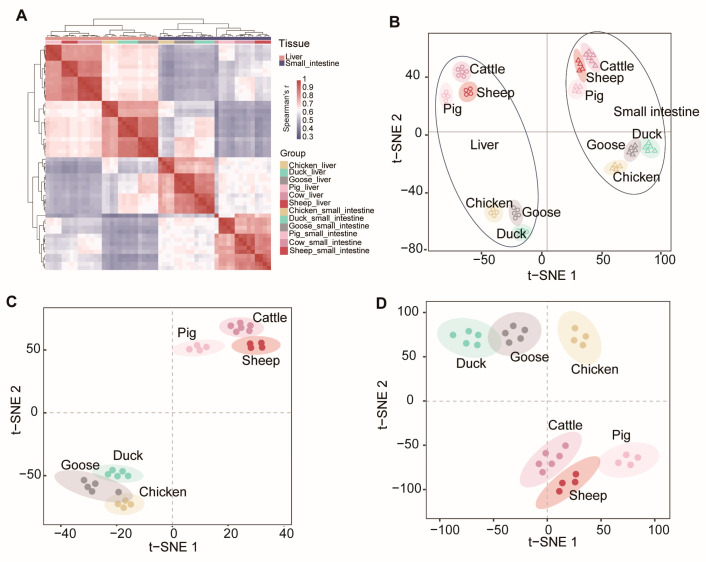
Global pattern of gene expression patterns. (**A**) Hierarchical clustering of samples by gene expression. Average linkage hierarchical clustering was used with the distance between the samples measured by Pearson’s correlation between the vectors of expression values. t-SNE (t-distributed stochastic neighbor embedding) of gene expression levels of all (**B**), liver (**C**), and small intestine (**D**).

The results showed that 56 samples were grouped into two broad clusters of mRNA expression profiles, with one cluster representing liver tissue and the other representing the small intestine (Figure 3A). Within these broad clusters, the mammalian samples were further divided into omnivores (pigs) and ruminants (sheep and cattle), while the avian samples were divided into chickens and anseriformes (geese and ducks) (Figure 3B). The t-SNE analysis of the liver (Figure 3C) and small intestine (Figure 3D) samples also showed that mammalian samples were clustered together. This observation supports the idea that gene expression changes evolve together with genetic variation over evolutionary time, resulting in lower expression divergence between more closely related species [24,25].

### 3.4. Differentially Expressed Genes in Two Tissues among Six Species

**Figure 4:**
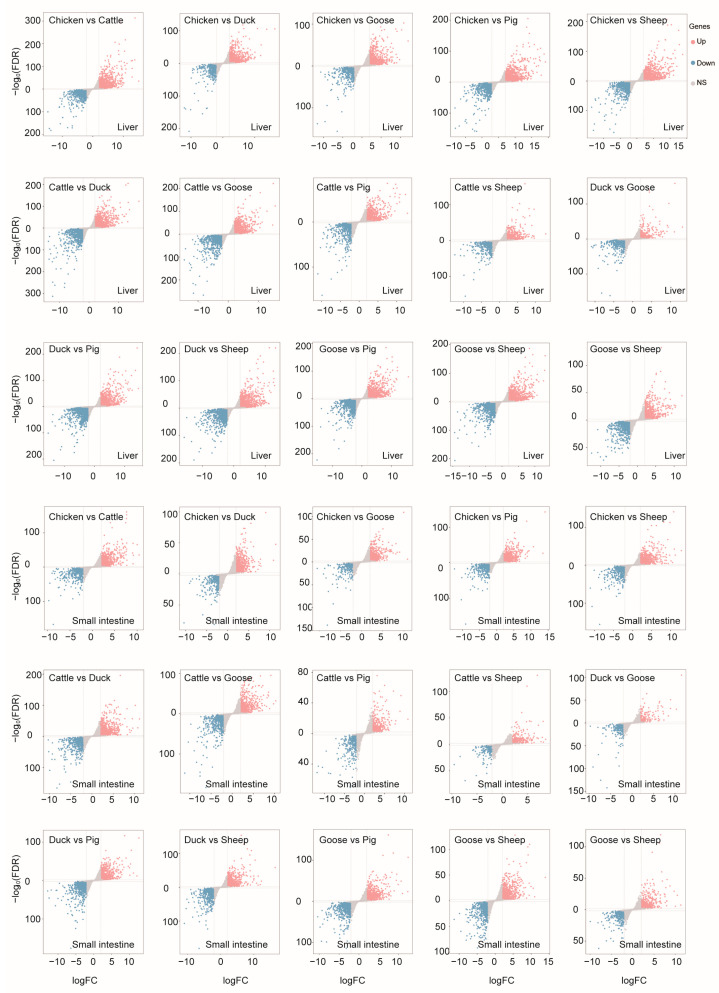
Differentially expressed genes detected in each comparison between each two species of liver or small intestine.

To evaluate differentially expressed genes (DEGs) among different species, we compared the expression levels using a threshold of Fold Change (FC) ≥ 2 or ≤0.5, along with a Bonferroni-adjusted *p*-value ≤ 0.05 (Figure 4).

**Figure 5:**
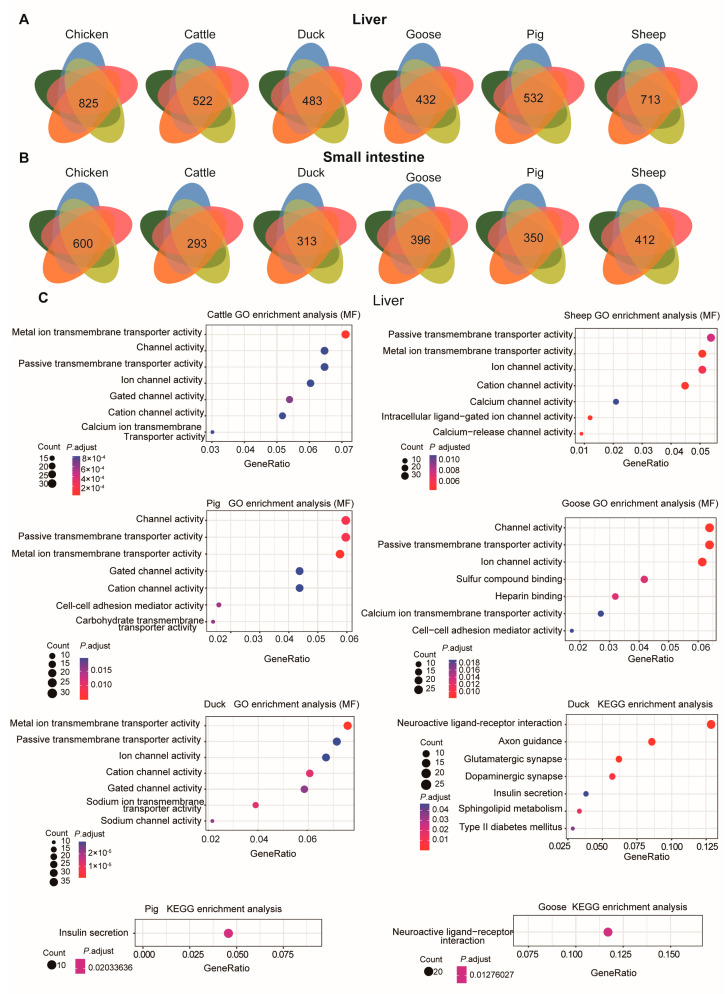
DEGs and their function in each species. The overlapping number of DEGs for the comparison between each species and each of the other species is shown in each Venn diagram. Venn diagrams illustrating DEGs detected in the liver (**A**) and small intestine (**B**) for each species. (**C**) GO enrichment analysis (molecular function, MF) (the first five panels) and KEGG pathway enrichment (the last three panels) for DEGs in the liver for each species using the overlapping DEGs.

We next examined the DEGs for each ; for example, for sheep, we compared sheep transcriptomic data with each of the other species and identified the overlapping DEGs. In the liver, there were 522, 825, 493, 647, 532, and 713 overlapping DEGs for cattle, chickens, ducks, geese, pigs, and sheep, respectively (Figure 5A). In the small intestine, there were 283, 600, 313, 496, 350, and 412 overlapping DEGs for cattle, chickens, ducks, geese, pigs, and sheep, respectively (Figure 5B). 

These DEGs, when compared across species, were mainly involved in channel and transporter activity in the liver (Figure 5C). For instance, the DEGs were mainly associated with the GO molecular function “Passive transmembrane transporter activity”. Only the DEGs in ducks, pigs, and geese were found to be enriched in KEGG pathways. In ducks, the enriched KEGG pathways included “Type II diabetes mellitus”, “Sphingolipid metabolism”, “Insulin secretion”, “Dopaminergic synapse”, “Glutamatergic synapse”, “Axon guidance”, and “Neuroactive ligand-receptor interaction”. In pigs, the enriched KEGG pathway was “Insulin secretion”, with genes such as *KCNJ11*, *ADCYAP1R1*, *ADCY3*, *KCNMB4*, *GLP1R*, *CHRM3*, *ADCY2*, *GCK*, *ADCY8*, and *ITPR3*. For geese, the enriched pathway was “Neuroactive ligand-receptor interaction”, including genes such as *NTSR1*, *TACR1*, *CHRM4*, *SCTR*, *GRIK3*, *GABRB3*, *GABBR2*, *GRID1*, *HTR1B*, *PMCH*, *NPY2R*, *CRHR2*, *EDN1*, *ADCYAP1R1*, *GRM7*, *PTGER2*, *QRFPR*, *CHRNA3*, *GLP1R*, and *LEPR*. GO enrichment analysis (BP) of these DEGs in the liver indicated they were mainly involved in “Synapse organization” and “Axon development” GO terms (Figure S2).

**Figure 6:**
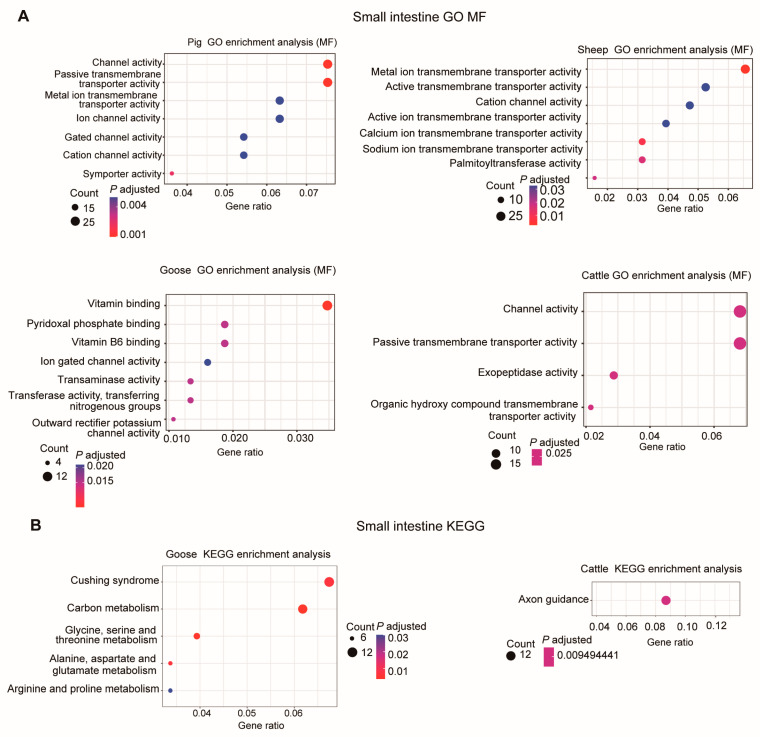
GO enrichment analysis (molecular function, MF) (**A**) and KEGG pathway enrichment (**B**) of DEGs in the small intestine for each species using the overlapping DEGs.

In the small intestine, the DEGs were primarily involved in “Channel activity”, “Metal ion transmembrane transporter activity”, and “Vitamin binding” GO molecular function terms (Figure 6A). For pigs, the DEGs were associated with “Cellular amino acid catabolic process”, “Sodium ion transmembrane transport”, “Sodium ion transport”, and “Organic acid catabolic process” GO-BP terms (Figure S3). 

Only for geese and cattle were enriched KEGG pathways detected (Figure 6B). In geese, these DEGs were involved in “Alanine, aspartate and glutamate metabolism”, “Glycine, serine and threonine metabolism”, “Carbon metabolism”, “Cushing syndrome”, and “Arginine and proline metabolism” KEGG pathways. In cattle, only the “Axon guidance” pathway was found to be significantly enriched (Figure 6B).

### 3.5. Correlation Analysis between Gene Expression and BA Content

**Figure 7:**
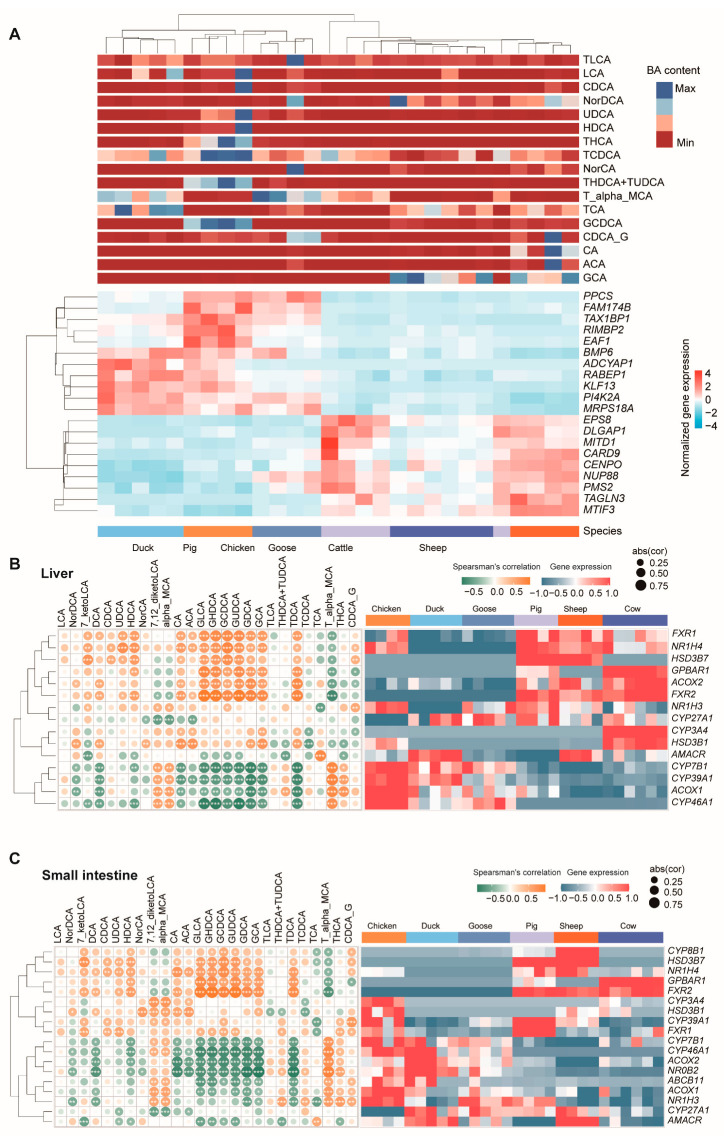
Correlation analysis between gene expression and BA content. (**A**) The top 20 genes most significantly associated with BA content. Heatmap of the expression of genes in the liver (**B**) and small intestine (**C**) responsible for bile acid composition. Left panels are the complete linkage clustering of genes based on Spearman’s correlation coefficient profiles, which were defined as the set of Spearman’s correlation coefficients calculated between the gene expression and the BA content of a sample. Yellow tiles indicate positive associations between these BA contents and genes; green tiles indicate negative associations. *, **, and *** represent significant differences at *p* < 0.05, *p* < 0.01, and *p* < 0.001, respectively; abs (cor) represent the absolute value of Spearman’s correlation coefficient. Color key is indicated in the upper right corner. The right panels are heatmaps of the expression of genes.

To explore the correlation between gene expression and BA content, we calculated Spearman’s correlation coefficient for the expression of each gene with BA content. The top 20 genes most significantly associated with BA content were *PPCS*, *FAM174B*, *TAX1BP1*, *RIMBP2*, *EAF1*, *BMP6*, *ADCYAP1*, *RABEP1*, *KLF13*, *PI4K2A*, *MRPS18A*, *EPS8*, *DLGAP1*, *MITD1*, *CARD9*, *CENPO*, *NUP88*, *PMS2*, *TAGLN3*, and *MTIF3* (Figure 7A, Table S7).

In addition, many genes were found to be responsible for bile acid composition, such as the ones in bile acid synthesis (*CYP7*, *CYP8*, *CYP27*, etc.) [26,27,28], and *ABCB11* [29], *GPBAR1*, and *FXR* [30] genes in bile acid transport (both uptake and efflux transporters) [31,32,33]. We next checked the expression of these genes, and further calculated their correlation with BA content. The results showed that *CYP8B1* was only expressed in three mammal species and highly expressed in the liver (Figure 7B). Two *FXR* genes were expressed in three mammal species, with a relatively lower expression, and one *FXR* gene expressed in three poultry species showed a higher expression. We then checked the correlation of the expression of these genes with the BA content. These genes were positively or negatively correlated with one or more BAs. In liver, all *FXR1*, *FXR2*, *NR1H4*, *HSD3B7*, *GPBAR1*, and *ACOX2* were found to be significantly positively correlated with GDCA, GLCA, GHDCA, GUDCA, and TDCA content (Figure 7B). On the other hand, the expressions of *CYP7B1*, *CYP39A1*, *ACOX1*, and *CYP46A1* had significantly correlation with these BA contents. These genes showed similar correlation patterns with BA in the small intestine (Figure 7C).

## 4. Discussion

Bile acids have surpassed their traditional roles as lipid solubilizers and regulators of BA homeostasis to emerge as important signaling molecules [34]. Once considered mere dietary surfactants, bile acids are now recognized as critical modulators of macronutrient (lipid, carbohydrate, protein) metabolism and the systemic pro-inflammatory/anti-inflammatory balance [35]. They are synthesized in the liver from cholesterol and stored in the gallbladder. When food enters the small intestine, bile acids are released to emulsify fats, increasing their surface area to facilitate the action of pancreatic lipase enzymes [36]. This process breaks down fats into fatty acids and glycerol, which are then absorbed by the small intestine [37]. 

The liver continuously produces bile acids, and the small intestine plays a crucial role in recycling them back to the liver through enterohepatic circulation [38]. This intricate relationship between bile acids, the liver, and the small intestine is vital for maintaining optimal digestive health and nutrient absorption. Different species have varying concentrations of BAs, which influences their susceptibility to obesity and fatty liver disease. 

In this study, we detected BA content within six different species (chickens, ducks, geese, pigs, sheep and cattle). Considering the crucial role of the liver and small intestine in BA synthesis and function, we also explored their gene expression patterns and compared the DEGs for the liver and small intestine among these species.

When examining BA content in bile, pigs had the greatest and geese had the lowest amount of BA content per given volume. These bile acids undergo enterohepatic circulation through the small intestine, liver, and kidney. Among the analyzed 22 BAs, 10 (GHDCA, GCDCA, THDCA+TUDCA, TCDCA, THCA, GLCA, GUDCA, HDCA, LCA, and UDCA) showed a higher abundance in pigs than in the other five species. Dihydroxy bile acids (DCA, HDCA, CDCA, and UDCA) and trihydroxy bile acid (CA) were the major BAs identified in the pig liver [39]. However, unlike CDCA and UDCA, which are approved drugs for the treatment of gallstones, HDCA is not marketed for any medical condition [39]. Hyodeoxycholic acid (HDCA) is a secondary bile acid and a metabolite produced by intestinal bacteria. Although the relationship between the composition of porcine bile acid and non-fatty porcine liver has not been directly demonstrated by scientific evidence, the HDCA was found to have therapeutic effects on non-alcoholic fatty liver disease (NAFLD) in multiple mouse models [40] by inhibiting RAN-mediated PPARα nucleus–cytoplasm shuttling [41]. The other BAs notably present in pigs are also worthy of further study to explore their potential functions.

It is interesting that duck had the highest level of TCA (taurocholic acid). A study found higher levels of TCA in the serum of acute decompensated cirrhotic patients compared to those with compensated cirrhosis [42]. TCA is an active facilitator of cirrhosis progression, and therefore a potential therapeutic target for the prevention and treatment of cirrhosis [43]. Meanwhile, sheep and cattle showed similar concentrations of BAs in bile.

At the transcriptome level, consistent with BA content, mammal (pig, sheep and cattle) or bird (chicken, duck and goose) species showed similar expression patterns. Here, the DEGs in the liver were mainly enriched for the “Insulin secretion” pathway in pigs, with genes including *KCNJ11*, *ADCYAP1R1*, *ADCY3*, *KCNMB4*, *GLP1R*, *CHRM3*, *ADCY2*, *GCK*, *ADCY8*, and *ITPR3*. Due to its central role in the secretion of insulin, the inwardly rectifying potassium channel subfamily J member 11 (*KCNJ11*) gene is one of the essential genes for predisposition to type 2 diabetes (T2D) [44]. The pituitary adenylate cyclase-activating polypeptide (PACAP)-selective PAC1 receptor (PAC1R) is a member of the vasoactive intestinal peptide (VIP)/secretin/glucagon family of G protein-coupled receptors (GPCRs) [45], which play important roles in metabolic disorders. *ADCY3* is a pivotal gene in classical ketogenic diets, which are fundamentally high in fat content, moderate in protein content, and low carbohydrates, for the treatment of epilepsy [46]. Fluid and bicarbonate secretion is a principal function of cholangiocytes, and impaired secretion results in cholestasis. Cholangiocyte secretion depends on the peri-apical expression of the type 3 inositol trisphosphate receptor (ITPR3) [47]. Duodenal mucosal resurfacing with a GLP-1 receptor (GLP1R) agonist increases postprandial unconjugated bile acids in patients with insulin-dependent type 2 diabetes [48]. The enhanced expression of these genes and pathways in pigs might be correlated with the greater levels of BA detected in the bile.

In the small intestine, bile acids facilitate lipid digestion and absorption [47]. Primary bile acids undergo dehydroxylation by bacteria in the small intestine, forming the secondary bile acids deoxycholic acid and lithocholic acid, respectively [49]. In addition, the absorption of bile acids by intestinal epithelial cells influences the activation of intracellular receptors like the farnesoid X receptor (FXR) and the G protein-coupled bile acid receptor 1 (*GPBAR1*) [30]. There are two FXR genes (*FXR1* and *FXR2*) expressed in mammals, but only one (*FXR1*) expressed in poultry species. This process significantly impacts the liver’s production of bile acids, as well as the metabolism of glucose and lipids in small intestine. For *GPBAR1*, it was the only detected expression in the mammal species. These results showed the different metabolism and absorption levels of BAs among species. In addition, small intestine DEGs were enriched for “channel activity”, “metal ion transmembrane transporter activity”, and “vitamin binding”, which is consistent with the function of the small intestine. These ion channels and transporters are ubiquitously expressed on the cell membrane, and participate in a plethora of physiological process such as contraction, neurotransmission, and secretion, amongst others. They are of great importance for maintaining membrane potential homeostasis, which is essential to the absorption of nutrients in the small intestine [50]. For pigs, DEGs involved in the “cellular amino acid catabolic process” and “sodium ion transmembrane transport” indicate a potential specialization in amino acid metabolism and the regulation of ion balance.

## 5. Limitations of the Study

For the comparative transcriptome study, we used orthology gene sets to explore DEGs among species. We did not explore whether there are functional differences in the number and representation of genes between birds and mammals, or what percentage of the orthology of genes is involved in BA and lipid or other metabolisms. The liver is the leading site for metabolism, and the dysregulation of the hepatic lipid metabolism precipitates disorders, such as NAFLD, affecting the whole body [51]. Despite some studies finding that highly represented BAs like HDCA have therapeutic effects on NAFLD, it is worth noting that the expression of these regulated genes might also be affected by the dietary requirements of each species. In addition, the potential medicinal functions of the BAs which were highly expressed in pigs or other animals were not explored. Further studies are needed to explore these BAs’ functions and the regulated role of the genes in relation to BA’s metabolism for each species.

## 6. Conclusions

In conclusion, we detected the composition difference in BAs in six species; pigs showed a difference from other species, and contained a very high BA content. Additionally, our comparative analyses of DEGs across species provides a comprehensive view of the molecular underpinnings of species-specific physiological responses. These findings contribute to a better understanding of interspecies differences in metabolism, digestion, and disease susceptibility, and may guide future research in comparative physiology and medicine. The top 20 significantly associated genes identified by these analyses may serve as potential biomarkers or therapeutic targets for modulating BA levels and associated metabolic processes. Future studies should compare intestinal metagenomes, transcriptomes, and bile acid content in different species to find more genes and bacteria involved in bile acid metabolism.

## Data Availability

The datasets presented in this study can be found in online repositories. The name of the repository and accession number(s) are as follows: PRJNA1117571.

## Supplementary Materials

The following supporting information can be downloaded at: https://www.mdpi.com/article/10.3390/metabo14080451/s1, Figure S1: Relative standard deviation (RSD) of sample metabolome; Figure S2: GO Enrichment Analysis (BP) of DEGs in the Liver; Figure S3: Research on the DEGs function of pigs. Table S1: Metabolite calibration table; Table S2: Sample quantification calculation table; Table S3: Principal component analysis table; Table S4: Analysis of bile acid differences; Table S5: Comparison of bile acid content; Table S6: CDNA library of liver and small intestine from 56 samples; Table S7: The correlation between gene expression and BA content.

## Abbreviations

BA, bile acid; ACA, N-(p-amylcinnamoyl) anthranilic acid; CA, cholic acid; CDCA, chenodeoxycholic acid; CDCA-G, chenodeoxycholic acid-3-β-D-glucuronide; GCA, glycocholic acid; GCDCA, glycochenodeoxycholic acid; GDCA, glycodeoxycholic acid; GHDCA, glycohyodeoxycholic acid; GLCA, D-glucuronic acid; GUDCA, glycoursodeoxycholic acid; HDCA, hyodeoxycholic acid; NorCA, norcholic acid; NorDCA, 23-nordeoxycholic acid; T-alpha-MCA, tauro-alpha-muricholic acid; TCA, taurocholic acid; TCDCA, taurochenodeoxycholic acid; TDCA, taurodeoxycholic acid; THCA, tetrahydrocannabinolic acid; THDCA+TUDCA, taurohyodeoxycholic acid (THDCA) and tauroursodeoxycholic acid (TUDCA); TLCA, taurolithocholic acid; UDCA, ursodeoxycholic acid.
