# Supplementary material for: Bile Acid Composition and Transcriptome Analysis of the Liver and Small Intestine in Different Species
Source: Metabolites. 2024 Aug 15;14(8):451. doi: 10.3390/metabo14080451 (PMC11355998; doi:10.3390/metabo14080451)
Supplement: Supplementary file 1 [file metabolites-14-00451-s001.zip › Suplementary figures.pdf]

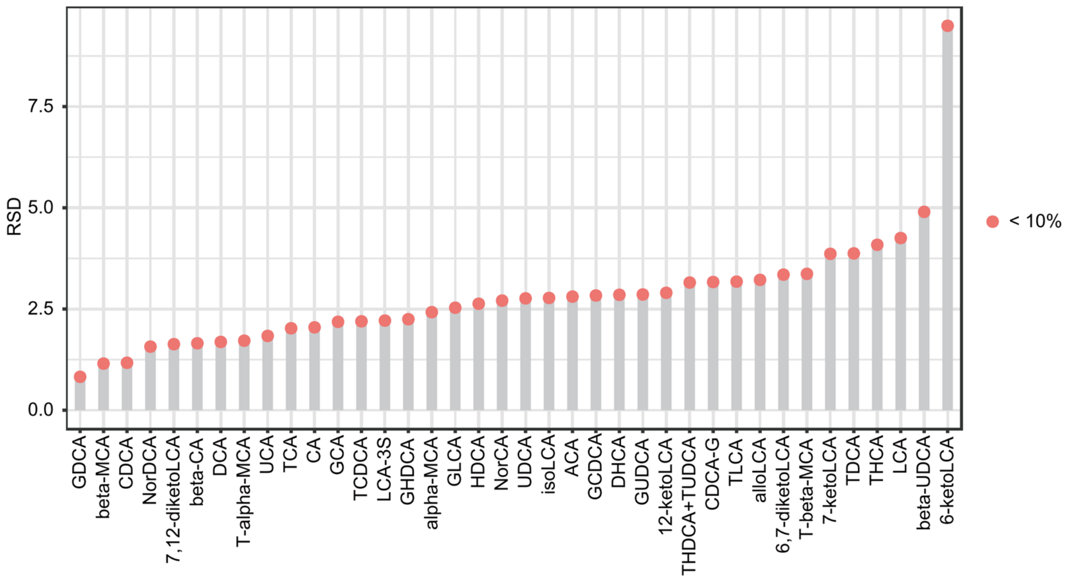

Figure S1. Relative standard deviation (RSD) of metabolites in all samples.

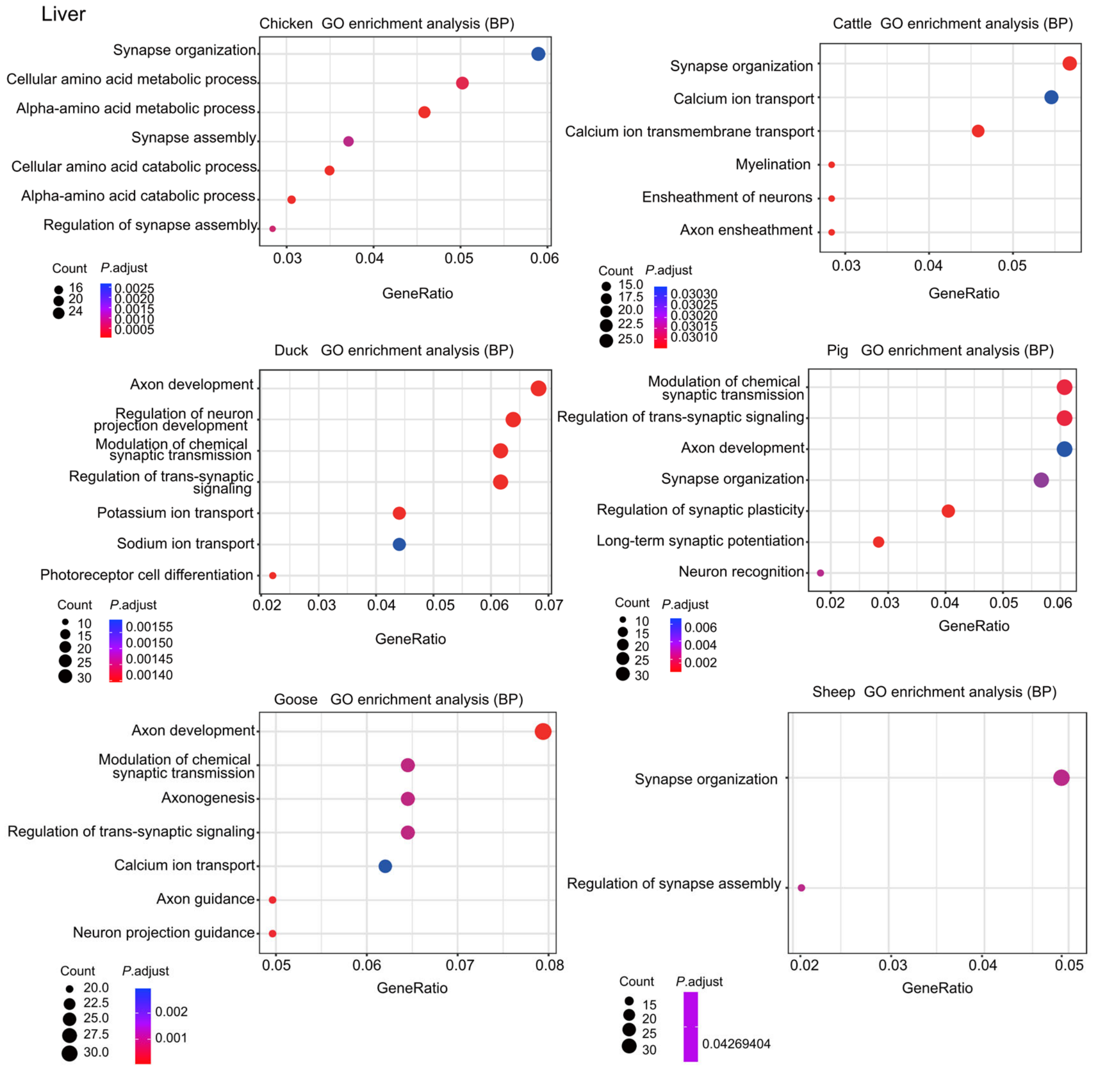

Figure S2. GO enrichment analysis (BP) of these DEGs in the liver.

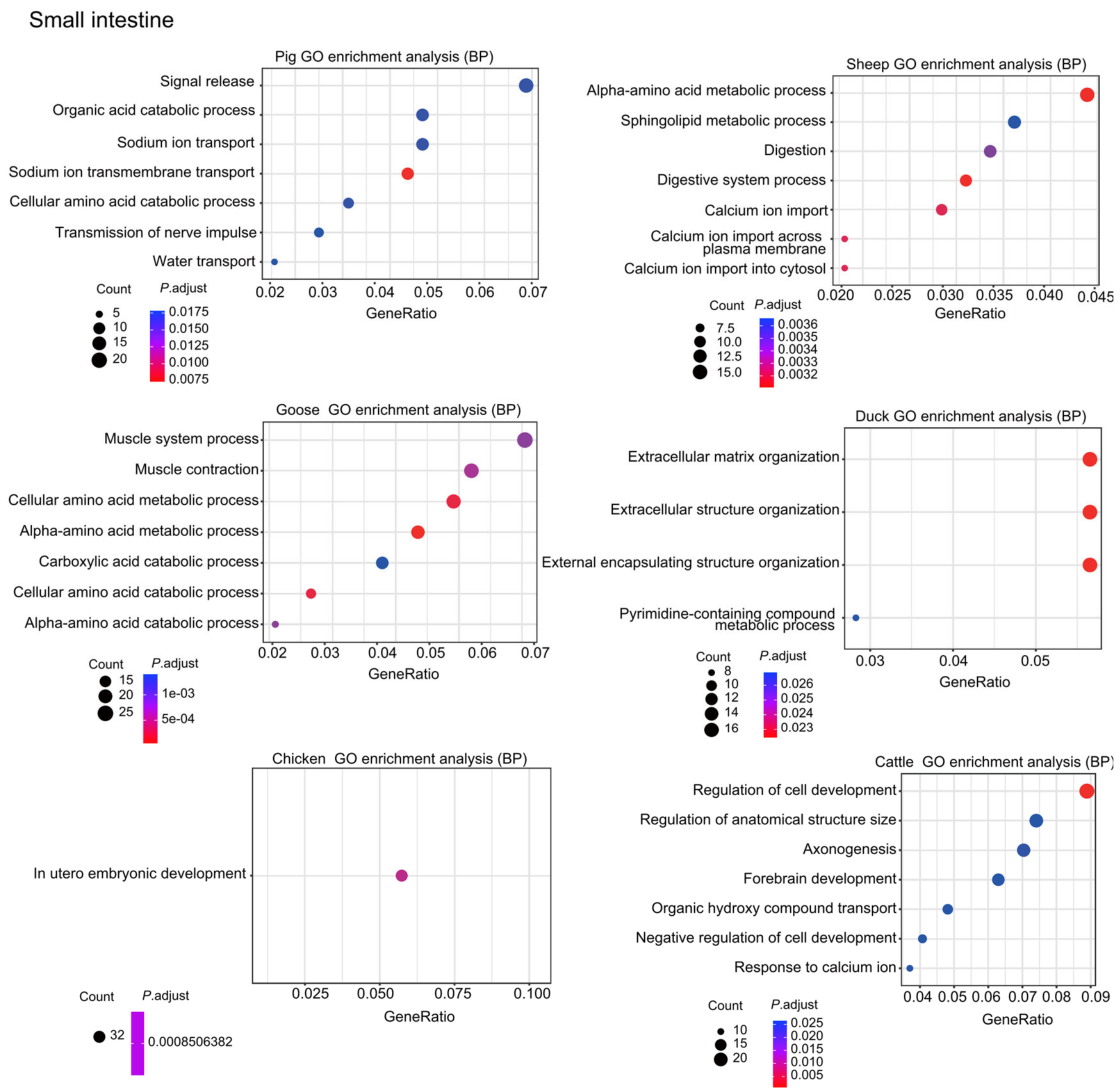

Figure S3. GO enrichment analysis (BP) of these DEGs in the small intestine.
